# Do I want to work from home today? Specific job crafting strategies of public service employees working from home during the COVID-19 pandemic in Germany: a qualitative study

**DOI:** 10.3389/fpsyg.2023.1183812

**Published:** 2023-10-13

**Authors:** Laura Seinsche, Kristina Schubin, Jana Neumann, Holger Pfaff

**Affiliations:** Institute of Medical Sociology, Health Services Research and Rehabilitation Science, Faculty of Human Sciences, Chair of Quality Development and Evaluation in Rehabilitation, Faculty of Medicine and University Hospital Cologne, University of Cologne, Cologne, Germany

**Keywords:** job crafting, job demands, job resources, working from home, job satisfaction, productivity, public service, COVID-19

## Abstract

**Background:**

After the outbreak of the COVID-19 pandemic, employees in Europe increasingly worked from home. In the German public sector, many employees experienced working from home for the first time. Concurrently, employees could use job crafting activities to alter job demands and resources while working from home. This exploratory case study aims to shed light on how public service employees craft their job demands and job resources, and how they perceive job satisfaction and productivity while working from home during the COVID-19 pandemic. A novel theoretical approach is applied to explore crafting activities that target specific job demands and resources when working from home, using a combined framework of resource-based job crafting based on the Job Demands–Resources model and time-spatial job crafting.

**Methods:**

Qualitative telephone interviews were conducted with employees from different public sectors in Germany between December 2021 and February 2022. According to the COREQ guidelines, the 12 semi-structured interviews were audio-recorded, transcribed verbatim, and content-analyzed using MAXQDA.

**Results:**

The results suggest that employees, who were new to working from home, developed personal crafting strategies for their flexible work environment. These strategies supported them in coping with hindering job demands (e.g., measures regarding work-related availability or interruptions) by optimizing their working conditions. Additionally, employees used strategies to increase their social resources (e.g., initiating meetings with colleagues) and structural resources (e.g., installing additional work equipment, planning of office days and working-from-home days). The use of given job resources and optimization of job demands are closely linked to the time-spatial demands fit. Thereby, the time-spatial demands fit is used to combine workplaces, work hours, or work tasks with the provided resources and demands to achieve an optimal work environment, which also facilitates employees' productivity and satisfaction.

**Conclusion:**

The results enrich the resource-based and time-spatial demand job crafting research by adding specific job crafting strategies utilized by public service employees. Furthermore, the results highlight job crafting strategies for enhancing job satisfaction and productivity when working from home in the post-pandemic world, thus offering valuable insights for researchers and practitioners.

## 1. Introduction

The COVID-19 pandemic has accelerated the transformation of work processes to a working-from-home environment in Europe (Eurofound, 2020). In turn, this leads to a clash of traditional work–life boundaries and new job demands and resources that employees have to face (Barbieri et al., 2021). Especially in the public service sector, working from home was newly established during the pandemic in Germany (Brenke, 2016; Siegel et al., 2020). Siegel et al. (2020) reported that before the COVID-19 pandemic in Germany, a mere 2% of public service employees worked from home for more than 50% of their working time. However, during the initial lockdown period, the number of remote workers increased to 34% working from home for 75–100% of their work hours. “Working from home, also called remote work (RW), telecommuting, teleworking, homework, home office, mobile work, outwork and the flexible workplace, is a work arrangement, in which employees do not commute to their workplace in the company” (Bellmann and Hübler, 2021, p. 424). As many organizations were not prepared for the switch to working from home and did not have established working policies (Carnevale and Hatak, 2020; Rudolph et al., 2021), employees had to reorganize and structure their work day by themselves. In consequence, the work environment became more flexible, corresponding with the “New Ways of Working”, where employees can decide when and where they work (van Steenbergen et al., 2018). A flexible working environment, encouraged by working from home or alternate work days from home, provides increased job autonomy that fosters proactivity and self-initiation (Saragih et al., 2021). In turn, job autonomy is a precondition for job crafting activities (Wang et al., 2021; Brauchli et al., 2022; Pijpker et al., 2022), meaning that employees adapt their approaches to work and modify certain job aspects. Job crafting is originally defined by Wrzesniewski and Dutton (2001) as “the actions employees take to shape, mold, and redefine their jobs (…) physically, by changing a job's task boundaries (…) cognitively, by changing the way they think about the relationships among job tasks, and what their job is relationally, by changing the interactions and relationships they have with others at work” (p. 180). Regarding the pandemic, job crafting strategies have been suggested as helpful to cope with job demands and resources (Kerksieck et al., 2022) since studies have shown that working from home increased job demands from employees' perspectives during the pandemic. The increased job demands were, e.g., work overload, time pressure, and cognitive and emotional demands (Ingusci et al., 2021; Wang et al., 2021). Hence, the increase in job demands during the COVID-19 pandemic may have led to increased anxiety and stress (Ingusci et al., 2021), if employees did not find proactive ways to cope with the situation. Thus, the consideration of job crafting strategies that can support balancing job demands and job resources in public service employees' work lives is important for research and practice (Nissinen et al., 2022).

Kniffin et al. (2021), who investigated the implications of COVID-19 and the workplace, suggest that future research should be carried out to determine whether job resources can still be effectively used by employees and to what extend job crafting can be as effective compared to pre-COVID-19. Especially during the pandemic, the chance to investigate full-time remote work, which has never occurred to this extent before, presented itself. In addition, 1 year after the outbreak of the pandemic, the social distancing measures were often reduced, thus giving the employees the choice to work from home or on-site. This is an advantage to analyze how time-spatial demand crafting takes place in actual work settings. In the context of organizational change, job crafting strategies have proven to be efficient in adapting to a new situation, reducing stressors, and making use of tools (Petrou et al., 2018; Vakola et al., 2019). Furthermore, job crafting activities may have become a necessity to deal with the job demands imposed on employees during the pandemic (Stempel and Siestrup, 2022).

Especially, in the public sector, research on job crafting is still scarce in comparison with the private sector (Audenaert et al., 2020; Luu, 2021). To the authors' knowledge, there are currently no studies in Germany that have investigated public service employees' job crafting strategies based on the Job Demands–Resources model and time-spatial demands fit from a qualitative perspective. We believe these perspectives have to be intertwined because employees decide where and when to work, based on the given demands and resources. However, there are a few studies focusing on job crafting during the COVID-19 pandemic from other countries in the public sector or partially in the German public sector (e.g., Ingusci et al., 2021; Kerksieck et al., 2022; Nissinen et al., 2022; Pijpker et al., 2022). There is one qualitative study that has evaluated strategies of public service employees to cope with working from home in Australia (Oakman et al., 2022).

Thus, this study contributes to the existing body of research on the effects of the COVID-19 pandemic on working conditions in Europe. In addition, this study provides insights into actual job crafting strategies and encourages further research in the field of general research on working from home and job crafting. The outcomes of crafting activities and the motives for crafting are comparably well-researched (e.g., de Bloom et al., 2020; Kujanpää et al., 2022), while we know little about the actual crafting strategies themselves. This exploratory research aims to shed light on specific crafting strategies that public service employees use to shape job demands and resources while working from home. Even if job crafting strategies have been studied before, previous studies do not provide practical guidance for employees to improve their working-from-home situation. Qualitative experiences can help to understand the real-world experiences of employees working from home and the development of practical supporting work strategies (Oakman et al., 2022). As Mäkikangas (2018) found out, employees can combine different job crafting strategies. Therefore, in this study, we focus on the (1) resource-based job crafting strategy by Tims and Bakker (2010) and Tims et al. (2012) aiming at the job resources and demands, which we believe to be important in the current context of extensive working from home. Additionally, we focus on two relatively new job crafting strategies that we believe to be important in the context of working from home: (2) time-spatial job crafting by Wessels et al. (2019) and (3) optimizing demands (Demerouti and Peeters, 2018).

We follow a novel approach, applying the concept of time-spatial job crafting in a qualitative case study. We argue that the COVID-19 pandemic has led to an intensive working-from-home experience, which is ideal for gaining more knowledge about time-spatial job crafting. Before, resource-based crafting has often been regarded in quantitative research (Tims et al., 2022), and we strive to add an integrative qualitative view by investigating which actual crafting strategies are used in the public service sector.

Therefore, we ask the following research questions:

1. How do public service employees craft their job demands and job resources when working from home?
2. How do public service employees perceive job satisfaction and productivity when working from home?

## 2. Theoretical foundation

This section briefly introduces the Job Demands–Resources theory and job crafting, including time-spatial job crafting, as a research frame of the current study. Additionally, the job demands and job resources in the public sector during COVID-19 are described.

### 2.1. Job crafting: a brief literature review

The general idea of job crafting is that in contrast to a job design perspective (Hackman and Oldham, 1980), where job characteristics are imposed on employees by management, employees can act as proactive job crafters. Tims and Bakker (2010) and Tims et al. (2012) have integrated job crafting research from a resource-based perspective in the Job Demands–Resources model (JD-R model) (Demerouti et al., 2001; Bakker and Demerouti, 2007). Therefore, employees can engage in different crafting strategies to increase their person-job fit: (1) increasing structural job resources (e.g., developing their skills and increasing decision latitude), (2) increasing social job resources (e.g., asking colleagues for help), (3) decreasing hindering demands, or (4) increasing challenging demands (Tims et al., 2022). Examples of the strategies aiming at increasing job resources are self-initiated learning of new skills or asking colleagues for help, while challenging demands can be increased through voluntary participation in new tasks (Tims et al., 2022). Hindering job demands can be interruptions, e.g., necessary work-related availability to colleagues. Thus, employees could employ a strategy such as assigning certain time frames to check their emails (Op den Kamp et al., 2018). This framework has been extended by Demerouti and Peeters (2018), who added the strategy of optimizing demands (aiming at making work more efficient). Optimizing demands means simplifying procedures and eliminating obstacles (Costantini et al., 2021), which is opposed to the strategy of minimizing hindering demands, making work less intense. Since cognitive crafting is only considered in the original approach, a fundamental difference exists between the original crafting approach of Wrzesniewski and Dutton (2001) and the resource-based crafting approach (Tims et al., 2022). Later on, other propositions were introduced to classify job crafting strategies and overcome the isolated perspective of both approaches (e.g., Lichtenthaler and Fischbach, 2019; Zhang and Parker, 2019).

In previous research, goals and antecedents of job crafting were often mixed (Kujanpää et al., 2022) leading to an integrative needs model of crafting by de Bloom et al. (2020), who link job crafting strategies to psychological needs satisfaction. The framework shows the various dimensions and the different aims of job crafting that have been studied. For example, the motives of job crafting have been studied as the antecedents of job crafting strategies (e.g., Slemp and Vella-Brodrick, 2014), as well as the context of blurring boundaries (e.g., Gravador and Teng-Calleja, 2018) or leisure crafting (e.g., Demerouti et al., 2020). One of the crafting strategies situated at the interface of work and home is time-spatial job crafting (Wessels et al., 2019), which is fitting for flexible work environments, in which employees need to match their tasks and private demands to working hours and workplaces. The authors define “time-spatial job crafting, where employees make active changes to their work, relating to working hours, places, and locations of work” (p. 5). This type of job crafting consists of different levels, namely, reflection, selection, and adaption. At first, employees need to be aware of their private demands and work tasks. Then, they can actively select working hours and workplaces fitting both demands. Finally, they may adapt the working hours and work location or private demands and tasks to optimize the time-spatial demands fit. It is proposed that employees who engage in time-spatial crafting activities may retain their productivity, work–life balance, and engagement on a daily level (Wessels et al., 2019).

Job crafting strategies as well as their antecedents and outcomes have been extensively researched (for an overview, see Rudolph et al., 2017; Lichtenthaler and Fischbach, 2019; Zhang and Parker, 2019). In general, job crafting has been linked to numerous positive outcomes for employees and employers (see de Bloom et al., 2020; Tims et al., 2022). For employees, job crafting can result in wellbeing and job satisfaction because there is a better fit between personal goals and work (Tims et al., 2013). Other outcomes that have been linked positively to crafting activities are performance (Boehnlein and Baum, 2020; Petrou and Xanthopoulou, 2020), work engagement (Demerouti and Peeters, 2018; Frederick and VanderWeele, 2020), work satisfaction (Boehnlein and Baum, 2020), and employee wellbeing (e.g., Tims et al., 2013; also on a weekly level, Petrou et al., 2017). Furthermore, job crafting behavior has a protective potential for employees' mental health (Uglanova and Dettmers, 2022) due to the reduction of psychological distress (Sakuraya et al., 2016), burnout (Tims et al., 2013; Singh and Singh, 2018), and exhaustion (Petrou et al., 2015; Shi et al., 2021). Especially in the context of telework, a positive influence of job crafting was found to enhance performance (Liu et al., 2021).

### 2.2. Job demands and resources during COVID-19

The COVID-19 pandemic has led to an increase in working from home full time, for which many organizations were not prepared (Carnevale and Hatak, 2020; Rudolph et al., 2021). It imposed a new situation for public service employees and employers. Thus, job demands and resources must be re-evaluated because they may be alternated in a way that destabilizes the existing balance of job demands and resources.

Studies focusing on the impacts of remote work have yielded mixed results (e.g., Gajendran and Harrison, 2007; Oakman et al., 2020). On the one hand, job resources can be increased such as autonomy (Sardeshmukh et al., 2012; Tavares, 2017), while other important resources such as social resources may be decreased because social interaction and contact are reduced (Sardeshmukh et al., 2012; van Steenbergen et al., 2018). Therefore, working from home is associated with increased isolation (Tavares, 2017; Dettmers and Plückhahn, 2022) due to the reduced contact but simultaneously linked to positive health outcomes due to experienced job autonomy and time flexibility (Sardeshmukh et al., 2012; Garcia-Contreras et al., 2021). On the other hand, job demands can decrease when working from home, since distractions or interruptions by others are reduced in comparison with working on-site (van Steenbergen et al., 2018), and time pressure decreases (Sardeshmukh et al., 2012). Reduced hindering demands can have positive outcomes as they decrease work-related stress and pressure (Tims et al., 2013). However, newly imposed demands can result from increased workload (Wu and Chen, 2020), blurred boundaries, and an increased work–home conflict leading to work exhaustion (Palumbo, 2020).

As the abovementioned interplay of changed demands and resources points out, various studies have shown working from home can increase affective wellbeing (Anderson et al., 2015), work satisfaction (Hornung and Glaser, 2009; Troup and Rose, 2012; Bae and Kim, 2016), and performance (Vega et al., 2015). Other studies have shown that working from home can decrease stress (Major et al., 2008; Hayman, 2010)—as well as reduce stress associated with commuting (Filardi et al., 2020)—and increase energy levels (Major et al., 2008) and quality of life (Hornung and Glaser, 2009; Filardi et al., 2020). In contrast, an increase in stress and pressure was also found, leading to a loss of productivity (Wu and Chen, 2020).

## 3. Materials and Methods

### 3.1. Study design

Case studies enable addressing questions of “how” and “why” (Yin, 2003) and offer an understanding of the contextual factors that influence participants' behaviors. Since we applied a qualitative approach to answer our explorative research questions, we followed the interpretivist paradigm stating that reality is socially constructed because individuals attach meaning to their experiences and the social world consists of their perceptions based on this construction (Wilson, 1973; Neuman, 2003). Hence, problem-centered interviews were chosen to capture the social reality related to subjective perceptions, individual actions, and ways of processing a certain experience with an unbiased approach (Witzel and Reiter, 2012). An exploratory approach was deemed appropriate due to the limited research on the topic (Stebbins, 2001; Mayring, 2018; Rendle et al., 2019) and can provide valuable in-depth insights into public service employees' experiences of working from home (Kuckartz, 2014).

To ensure a systematic research process despite the exploratory approach, we followed established qualitative data analysis procedures (Kuckartz, 2010), as well as quality criteria and reporting guidelines (Tong et al., 2007). The completed checklist was applied to ensure the quality of the data collection, analysis, and reporting (Supplementary material 1). The same sample and procedure were also used in our article analyzing job demands and job resources of public service employees during COVID-19 (Seinsche et al., 2022). The study design and methodology were approved by the ethics committee of the University of Cologne, and the study was prospectively registered (Ref No. 21-1417_1).

### 3.2. Sampling procedure

We used a purposeful sampling strategy (Patton, 2002) to identify the possible interview partners. All of the participants had previously completed two online surveys in a quantitative study regarding the topic of working from home. During the second wave of the study, they agreed to interview on their personal experiences and provided their email address (Neumann et al., 2021). Thereafter, public service employees were contacted and asked to provide additional information on their job characteristics and demographic details. Hence, the provided details could be used to facilitate a heterogeneous sampling strategy aimed at identifying different cases. The use of heterogeneous sampling based on criterion-oriented case selection is particularly suitable for developing theories or exploring the variability of the research object (Schreier, 2011). The sampling strategy aimed to obtain maximal variance in gender, age, leadership position, and current job position. Other selection criteria were the duration of working from home in the agency and participants' perception of the effectiveness of working-from-home implementation in their agency. There was one major exclusion criterion regarding participants' ability to work from home. Employees who could not or only partially complete their tasks at home were excluded from the sample. In sum, 20 potential interviewees were contacted via email with an invitation to the telephone interview. In line with the tailored design method by Dillman et al. (2014), the potential participants received up to three reminders, if they had not responded to the first or following emails. Ultimately, five contacted persons did not respond to our recruitment attempts, and three others dropped out later leading to less variety in the final sample than anticipated. These three possible interviewees, who had agreed on a meeting, dropped out before the interview took place due to health issues or family reasons.

### 3.3. Data collection

The semi-structured interview guideline was developed with the SPSS principle by Kruse (2015). To carry out this process, three researchers (JN, KS, and LS) collaborated to brainstorm potential questions based on a prior quantitative study on working from home in the German public sector (Neumann et al., 2020) and the job demands and job resources theory. In the next step, the questions were optimized regarding qualitative research standards (e.g., openness and suggestiveness). Finally, the questions were assembled into four topics of which each contained a narrative impulse and sub-questions (Helfferich, 2011). The final interview guideline with guiding questions and sub-questions can be found in Supplementary material 2.

Three female researchers (LS, KS, and JN) conducted qualitative interviews with the participants. All of them possessed a master's degree and had experience with qualitative data collection. Since all of the public service employees had participated in two online surveys before the qualitative data collection, they already knew about the study and showed an ongoing commitment. The three researchers communicated with the participants via email regarding the details of the scheduled interview. The telephone interview was the first interactional contact between the interviewees and the research team. In accordance with German data protection laws, written consent was obtained from each participant before the telephone call. The participants provided their signed consent forms via email. Due to COVID-19-related social restrictions, interviews were carried out over the telephone from December 2021 to February 2022. Before the telephone interview started, the interviewer briefly introduced herself and the aims of the study and its confidentiality. Participants were asked if they had any remaining questions and still wanted to participate in the interviews. After they repeated their consent, the audio recording started. During the interview, only the research team was present. The semi-structured guideline was handled as a flexible framework, where the interview questions ensured that the main research topics were included in the discourse and guaranteed a certain comparability between the 12 interviews (Patton, 2002; Witzel and Reiter, 2012). Nevertheless, the order of the questions remained flexible giving the interviewees a chance to include their own topics and maintain a natural narrative flow. The participants were encouraged to reflect on their typical workday experiences while working from home. Following the interviews, additional questions were asked to gather information to complete sample characteristics, such as number of employees in the agency and years of employment. The 12 interviews lasted from 26 to 60 min. During the interview, field notes were taken by the researchers. These provided additional guidance during data analysis when the context of the data could lead to more than one possible interpretation.

### 3.4. Participants

A total of 12 employees in the public service sector from different agencies in Germany were interviewed, with a mean age of 54.3 years (range: 29–62 years) and 33% of them being women. Their work experience in the current agency varied from 1 to 36 years, with a mean of 14.8 years. Before the COVID-19 pandemic, only a minority of participants (33.3%) had experience working from home. On average, they worked from home for 2.25 days during the time of data collection, whereas five participants worked from home every workday. It is worth noting that all participants reported having a designated workspace when working from home. Seven (58.3%) of the 12 participants held leadership positions. Table 1 contains the details of all participants.

**Table 1:** Characteristics of the interviewed public service employees.

**Interview**	**Age**	**Gender**	**Field of agency**	**Number of employees in the agency**	**Work experience in agency (yrs.)**	**Leadership position**	**Start of WFH**	**WFH amount (days/week)**	**Fixed workplace (WFH)**
1	59	m	Building and real estate	ca. 180	31	n	March 2020	5	y
2	61	f	Construction industry	4.000	28	y	2016	5	y
3	60	f	District governance	7 (in unit)	14	y	March 2020	0 (before 4–5)	y
4	50	m	Data protection	ca. 175	3	y	before COVID-19	3	y
5	62	f	Social welfare	1.100	36	n	Spring 2020	ca. 4	y
6	53	m	IT service	300 (in department)	2	n	March 2020	5	y
7	62	m	Building and property management	2,000	16-17	y	ca. 2009	1–3	y
8	49	m	Environmental management	1,200	20	n	March 2020	3–4	y
9	29	f	Learning and education	1,000	1	n	April 2021	4–5	y
10	56	m	Information and statistics	300	10	y	March 2020	5	y
11	55	m	Customs	10 (in unit)	12	y	2019	4	y
12	55	m	Telecommunication	3,000	4	y	March 2020	5	y

WFH, work from home.

### 3.5. Data analysis

The audio-taped files were transcribed verbatim following the rules by Dresing and Pehl (2018) and pseudonymized by a transcription service. The two authors (LS and KS) coded the data with the support of the software MAXQDA 2022 (VERBI GmbH, Berlin, Germany) (VERBI Software, 2022). The data were analyzed using the qualitative content analysis developed by Kuckartz (2010), which allows the combination of a deductive and inductive approach. Theory triangulation was used to create a framework based on the presented theories about resource-based job crafting research (including optimizing demands) and time-spatial demands fit to deduce the subsequent research questions. On this basis, deductive categories were developed. For example, the main categories of resource-based job crafting included increasing structural resources, increasing social resources, increasing challenging job demands, and decreasing hindering job demands. In the first coding round, the first author (LS) coded the interview material with deductive and inductive categories. The inductive codes were based on new themes that the participants included during the interviews. The second author (KS) coded one-third of the material with the established deductive categories and derived inductive categories from the material as well. After the first coding round, the results were discussed to develop a universal category system. The first author (LS) applied the new coding system to all of the interviews and the results were re-checked by the second author (KS) during the second coding round. The two authors (KS and LS) discussed and agreed upon the final coding framework, and data saturation was confirmed (Saunders et al., 2017). In this relation, data saturation refers to the saturation of the categories as for the coded interview material, no new information was detected, which concludes that within the limitations of the case study, the relevant aspects were covered. Afterward, the first author coded all transcripts by applying the final coding scheme, which comprises theoretical dimensions, main categories (based on the theoretical framework), and inductive sub-categories. The final coding scheme with definitions and illustrative quotes is available in Supplementary material 3. It entails representative quotations for each code to ensure inter- and intra-rater reliability (Helfferich, 2011). The first author (LS) translated the quotations from German to English, and the translated quotes were revised by the second author (KS). The transcripts were not returned to the participants for commentary, but a fact sheet with an overview of the findings was sent to the interviewees in autumn 2022. One of the participants thanked the authors for the provided results, but none of them gave additional feedback on the findings.

## 4. Results

**Figure 1:**
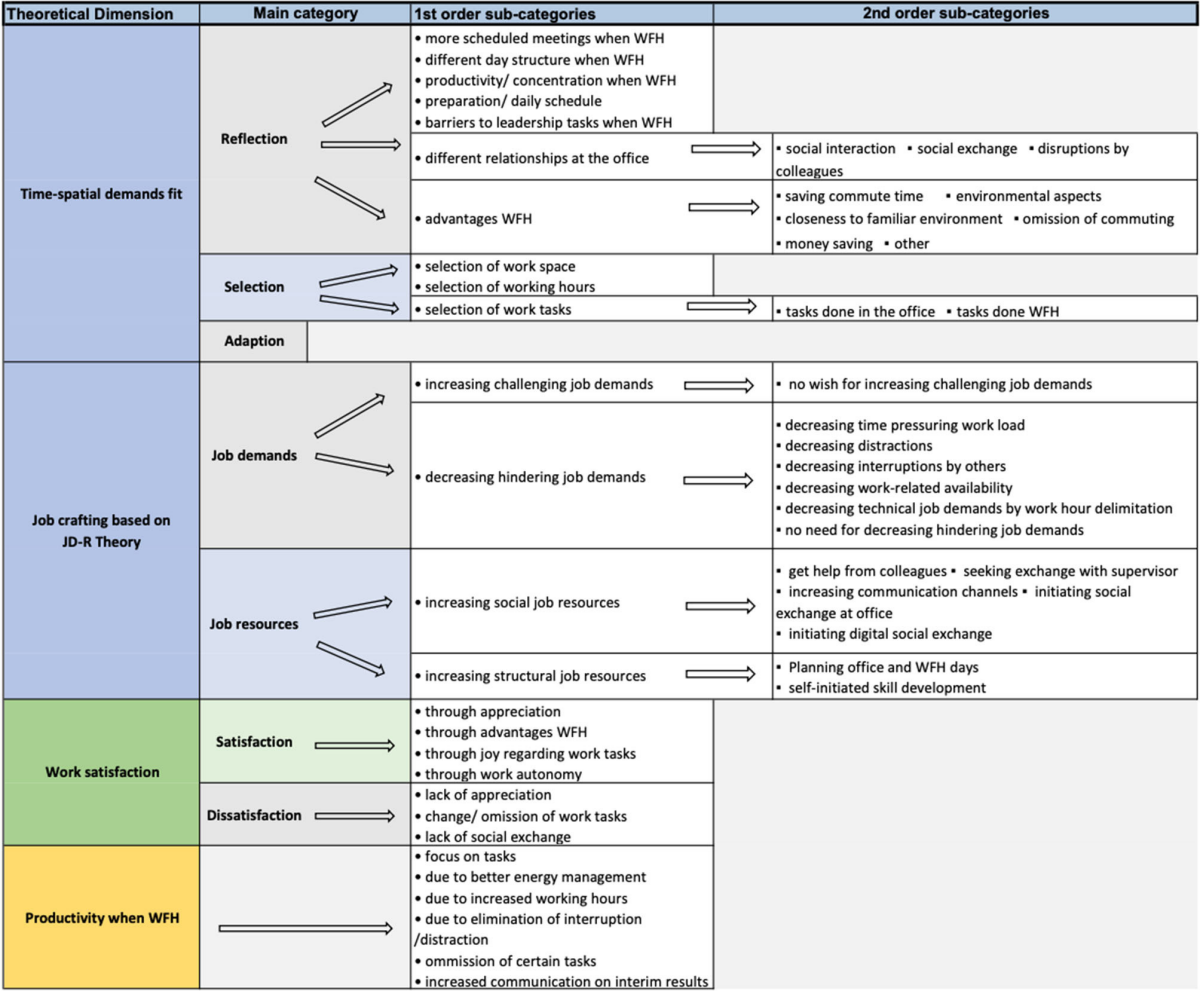
Overview of categories.

An overview of the categories and sub-categories is provided in Figure 1. All quotations, that are referred to, are provided in the Supplementary material 3. In the following, the job crafting strategies will be presented according to the corresponding theoretical frameworks. Additionally, the outcomes of the job crafting strategies and choice of work style will be described.

For the category “increasing challenging job demands”, the results suggested job enrichment of facility managers. These results were interesting because due to the COVID-19 pandemic, this group stayed on-site and received more responsibility and an upgrading of their job. Nevertheless, we excluded these results from the study, since it was not an active job crafting strategy implemented by the employees. Rather, this was imposed on them by the circumstances of the pandemic.

### 4.1. Job crafting strategies according to JD-R theory

#### 4.1.1. Strategies targeting job demands

Concerning the demands that were reported by employees, there were no employees who strived to increase challenging job demands. Employees' experiences rather pointed in the opposite direction. Thus, the sub-category was rephrased to “no wish for increasing challenging job demands”. One employee stated:

“*I am already working to capacity. So, I don't have to look for work or something like that.”* (interview 2)

On the contrary, many categories explain how employees decreased hindering job demands such as decreasing time pressuring work load (interview 6) and decreasing distractions at the office (e.g., by working from home, interview 4). There were also employees, who saw no need for decreasing distractions (e.g., loud phone calls of the husband at home) and were willing to “*put up with that”* (interview 5). Regarding interruptions by others, one of the employees suggested, when being interrupted at work:

“*It would be better to say, ‘Please make an appointment', but (laughs), yes, and that means I'm much more disturbed in my work processes.”* (interview 3)

The above suggestion is closely linked to decreasing the work-related availability which can be seen as a job demand. The participant also achieved to optimize this demand by scheduling video conferences (interview 3). Another crafting strategy targeting work-related availability entailed withdrawing from communication channels from time to time:

“*So, yes, there are different strategies, well um, if you're constantly online and constantly responding to your e-mails, you don't really get to work. You just have to find ways for yourself, maybe to structure it a bit.”* (interview 1)

One interviewee reported that he consciously selected time slots on the weekend for tending to his tasks because at this time point, hindering technical demands were lower:

“*That also happened to me once or twice—I don't want to say happened. That was a conscious decision, because it was simply easier for me to watch an import on Saturday, because if it had broken off, I would have had a lot more work on Monday than if I had intervened at the moment when it happened. In principle, I made it easier for myself at the beginning by switching on briefly. But that rarely happens.”* (interview 6)

#### 4.1.2. Strategies targeting job resources

From the employees' perspective, there was an emphasis on the increase of social job resources by enhancing the opportunities for social exchange, e.g., by increasing communication channels (interview 12) and initiating social exchange at the office or digital social exchange from home.

At the office, an open door can invite people to knock and come in (interview 3) and social interaction can be initiated by seeking out gathering places:

“*There you just go to the coffee room, or you go to your colleagues, who you now unfortunately only get into the house by phone or e-mail or chat or video conference, you used to very often say ‘I'll just go to my colleagues around the corner or one floor higher'.”* (interview 6)

This is why one interviewee stated “*we quickly came back to the office voluntarily for certain things, simply to see the colleagues.”* (interview 8).

When working from home, other measures have to be taken to start a social exchange, and making a phone call was perceived “*much harder than walking the three steps”* (interview 8) to the next office. Additionally, employees initiated digital coffee breaks, where everybody was invited to participate voluntarily and private talks could take place (interview 11).

According to one interviewee, while working from home, they implemented a practice of seeking assistance from colleagues on-site, who could forward documents via email (interview 8). Another strategy was to actively seek a conversation with the supervisor to negotiate their interests. One employee stated:

“*That's what I've learned over the course of time: Formulating my interests and then putting them forward, and then it works out well, yes.”* (interview 1)

There were also strategies reported that employees used to increase structural resources. These entailed the planning of office or working-from-home days, while, e.g., taking work documents home (interview 8) or deciding to go to the office to use the printer:

“*So, when I see that there are larger documents that I just don't want to work on, then I go to the office and then I stay there and then I print them out, among other things, and then I also stay in the office for the day.”* (interview 5)

Increasing structural resources also meant getting the right work equipment (e.g., office chair, interview 10) or a separate room for working from home (interview 11).

Employees were able to increase their resources through self-initiated skill development, e.g., by learning the software applications needed for extensive remote work (interview 7), using literature for guidance (interview 11), and learning to structure a working-from-home day:

“*I used to say, 'I'm not the kind of person who can do that, I need an orderly framework, I need the way to work so that I can say, 'Now I'm here, now I'm working, and when I close up here, I'll be gone again.' In this respect, it was a learning process for me to actually be able to structure myself.”* (interview 8)

### 4.2. Job crafting strategies according to time-spatial demands fit

Concerning the reflection of employees regarding their choice of workplace, working hours, and work tasks, the category “reflection” comprised the following sub-categories: “More scheduled meetings when WFH” (working from home), “different day structure when WFH”, “different relationships at the office”, “productivity/concentration when WFH”, “preparation/daily schedule”, “barriers to leadership tasks when WFH”, and “advantages WFH”.

First, scheduling appointments in the digital work environment seemed easier but more time-consuming (interview 9). Second, working from home allows for a different day structure. Thus, one employee stated:

“*I have a bit of a stomachache when I think about the possibility of having to work four days a week on site again, because that would mean a complete restructuring of everyday life for us or for me as an employee. And that goes hand in hand with a different work-life balance.”* (interview 9)

Additionally, employees reflected on the different relationships with colleagues at the office:

“*Of course, it's not the same as when I come into the office in the morning and greet my colleagues in person and you may run into each other and use this opportunity to talk about something on the fly.”* (interview 10)

Social exchange and interaction were different when they were able to meet in person. There was more engagement in small talk (interview 9) and more information passed on (interview 3). Simultaneously, the decreased chance for conversation led to fewer disruptions and a higher productivity or concentration on tasks (see also Section 4.3.2. productivity when WFH), especially, leading to distance imposed challenges as one of the supervisors recalled, when two employees bullied each other:

“*If I had been on the spot, I could have intervened and said ‘Stop, that's not your job'. (...) Since I was not there, I did not know what they were doing.”* (interview 2)

From the employees' perspective, the advantages of working from home were contemplated, when choosing one work form over the other:

“*And what I really like about working from home actually now, especially in winter, in the gray season, is the flexibility, that commuting is eliminated or at least partially eliminated. Thus, I can react more flexibly at the point. If it rains, I don't have to get on my bike and go to the office, but I can work from home.”* (interview 8)

The omission of commuting (interview 5) and saving commute time (interview 11) were considered reasons for working from home, whereas one of the employees missed this time “*because it was always something on the way to work, that you could concentrate a bit on the work already, so that you have mentally processed or prepared for it a little bit*” (interview 2). Instead, she used the time walking the dog in the morning for her mental preparation for work. Other aspects that were reflected were environmental factors (interview 7), money-saving (interview 1), and others such as the freedom of clothing (interview 5). The closeness to the familiar environment also played a role:

“*I experienced a great personal advantage, since during this Corona period I was able to watch my grandson grow up close to me. (...) We saw each other almost every day.”* (interview 1)

When it comes to the selection process of employees, they needed to decide on the tasks that were to be done and selected either tasks done working from home or tasks done in the office:

“*There are a few tasks that you can only do in the office. For example, signing off on invoices and the like, which we don't do electronically yet.”* (interview 5)

One employee reported that he has to “*use an app to book a workstation for the days*” (interview 4) on which he wants to work from the office because alternating workstations were implemented. The selection of the specific work location also depended on the right equipment:

“*And for me personally, I used the working from home option only very sporadically at the beginning, on one or two days a week, because it was simply a matter of using a private computer at home, sitting at the kitchen table, and all the problems that I'm sure you're familiar with and that a lot of people encountered, until the technical equipment was ready. That was the crucial point, does the equipment fit.”* (interview 8)

Employees enjoyed the flexibility of selecting working hours (interview 10). They may have “*logged off for an hour, then at 4 o'clock (..) simply resumed duty for an hour or an hour and a half”* (interview 1).

In terms of adaption, most interruptions of prior planned working processes were named:

“*I'm much more disturbed when I work at the office, right? I also make my daily schedule, but then I can't keep to it in part because I'm disturbed.”* (interview 3)

Additionally, getting used to continuous work–life blurring was mentioned as a form of adaption (interview 1).

### 4.3. Productivity and satisfaction as outcomes of job crafting strategies

In this section, the experiences of employees regarding the possible outcomes of working from home will be presented. As employees might have decided to work from home as part of the selection process and applied job crafting strategies, these are possible outcomes related to those choices. We already discussed potential health effects related to job demands and job resources (for details, see Seinsche et al., 2022).

#### 4.3.1. Satisfaction

Employees named several reasons for their job satisfaction that were not solely restricted to working from home. If employees experienced satisfaction, they named appreciation (interview 6) and joy regarding work tasks or field of work (interview 1) as reasons. Especially, the experienced advantages of working from home were reported as factors for satisfaction. One supervisor mentioned the reaction of his employees as working from home was allowed:

“*They're happy, they're so fully motivated, and those are also the cases, for example, where the work results, productivity and also the motivation have naturally shot through the roof.”* (interview 7)

The work autonomy linked to working from home is also reported by employees:

“*So, the flexibility is fantastic for me.”* (interview 11)

“*This relatively high degree of autonomy, I like that. And, um, I think that's also the most important point that I would say contributes to my satisfaction.”* (interview 1)

Factors regarding their dissatisfaction can be tied to the causes of the social distancing measures during the COVID-19 pandemic. Mostly, the lack of social exchange was reported:

“*Yes, one thing that I like about my work, but which is actually very limited now due to the pandemic, is the personal contact with colleagues in the various federal states. Or even internationally, because all of these face-to-face meetings have actually all been virtual conferences in the last two years. That is something that really makes up a large part of the attractiveness of the work, which has been lost as a result. That has to be said quite clearly, yes.”* (interview 10)

Another aspect that derived from the situation during COVID-19 measures was the change or omission of work tasks. Thus, employees were dissatisfied that certain components of their tasks such as traveling could not take place:

“*And on top of that, I also have to take care of certain administrative tasks in our team in connection with certain IT issues, and I'm not particularly happy about that. But that only has a limited connection with working from home. It's simply a shift of work tasks due to Corona, yes, where you then have no reason to postpone such things, because you don't have to go on an important business trip as an alternative, do you?”* (interview 5)

Finally, the lack of appreciation by supervisors was reported as a dissatisfying factor (interview 8).

#### 4.3.2. Productivity when WFH

Employees reported that they were more productive from their point of view when they worked from home. This perception was due to various reasons. First, they could better focus on tasks and concentrate on their work (interview 11). This was due to eliminated interruptions or distractions by colleagues (interview 9), as one employee stated:

“*I might have fewer disruptions there than in the office, especially when the office is busy again, and I can actually follow my own rhythm then.”* (interview 10)

Following their own rhythm led to better energy management. Therefore, one interviewee said:

“*Because I simply do some exercise at lunchtime and I somehow have the feeling that after lunch or after a lunch break, my brain switches off automatically, which is a bit of an exaggeration, but when I work from home I really start somehow fresher into the second half of the work day after lunch break.”* (interview 9)

Another aspect was that some work tasks were simply eliminated because of the digital work environment. Thus, employees saved travel time or preparation time for meetings, where the room previously needed to be prepared or coffee needed to be made for colleagues (interview 6). One of the interviewees also stated that he has worked more since the saved commute time was “*converted into office work”* and more tasks. Hence, he asked himself if he “*was always necessarily more effective”* (interview 8).

The digital communication also allowed for more increased communication on interim results, which may have prevented working for nothing, because errors in work processes could have been detected earlier:

“*Then we might have another meeting in the middle of the day or at the end of the day, where we say ‘This is the work progress now. Does this fit into the management concept?' and if it doesn't fit or if it fits, then it's good, then (...) efficiency increases and if it doesn't fit, efficiency also increases, because the misguided development hasn't continued over 14 days.”* (interview 12)

## 5. Discussion

This study contributes to the growing body of literature about the effects of working from home in Europe. First, the results provide insights into actual job crafting strategies that public service employees used 1 year and a half after the breakout of the COVID-19 pandemic. Second, it discusses how employees combine job crafting strategies to cope with job demands and increase resources. Third, our study attempted an integrative view of resource-based job crafting, optimizing demands, and time-spatial demands fit, and the theoretical relations will be discussed. Finally, possible impacts of crafting strategies and working-from-home conditions are reported from employees' point of view. In the following, the research questions are answered.

**1. How do public service employees craft their job demands and job resources when working from home?**

The results indicate that public service employees craft their job demands and job resources when working from home corresponding to the resource-based job crafting theory by Tims and Bakker (2010) and Tims et al. (2012). Most of the material was coded in regard to increasing social and structural resources as well as optimizing demands. Similarly, Nissinen et al. (2022) reported to have found all job crafting dimensions in their study of public service employees, while increasing structural job resources was the most often reported dimension.

The fact that employees shied away from seeking challenging demands could also be explained by the uncertainty of the work situation during the COVID-19 pandemic and the increased job demands placed on employees, e.g., through the extensive use of technology and digital communication channels (Ingusci et al., 2021). Therefore, we found evidence of how public service employees use different strategies aiming at decreasing hindering job demands. Mainly, they optimized demands to make work more efficient (Demerouti and Peeters, 2018). Strategies that aimed at decreasing work-related availability (e.g., by scheduling online appointments or withdrawing from communication channels) supported employees because they made demands more predictable and interruptions in work processes were limited. Regarding the increase of resources, public service employees reported different strategies to increase social resources. When on-site, the door of the office can be kept open, while digital coffee breaks can help to initiate more social exchange in an online work environment. In addition, increasing structural resources was named as a useful strategy.

Concerning the time-spatial demands fit by Wessels et al. (2019), employees reflected on the disadvantages and advantages of working from home and on-site. For example, employees reported that they changed their day structure when working from home. Thus, they adapted to their new working-from-home environment and implemented new lifestyles. This may make an additional adaption necessary when the work day structure changes after the pandemic. Regarding the category “adaption”, the challenge of “getting used to work life blurring” or interruptions of work processes played a role. During the coding process of the interviews, there were particularly few codes in the sub-category “adaption”. This may be explained due to the fact that the interviewees were invited to reflect on their regular working-from-home days. Thus, they often reflected on the advantages and disadvantages of the office or working-from-home environment. The reported results were more fitting to the sub-category “reflection” as they answered their preferred choice of work style.

The crafting strategies that optimized demands and increased resources relate to the time-spatial demands fit by Wessels et al. (2019) since the choice of where to work is closely linked to available resources and imposed demands. Stempel and Siestrup (2022) reported that job crafting activities were not found in newcomers to working from home, because they remained in an initial orientation phase and the use of job crafting strategies has to undergo an adaption process. This view corresponds to the proposition of Wessels et al. (2019) regarding time-spatial job crafting, where reflection of the time, space, and tasks has to take place first to select the best available work option. From the qualitative results, it can be derived that employees reflect on different aspects of the workplace, such as concentration, day structure, or advantages of working from home, and select their place to work based on the provided advantages of the workplace (home or office), work hours, and tasks. Shao et al. (2021) investigated the choice to work from home or on-site during the COVID-19 pandemic and identified technology stressors such as malfunction or lack of access to the software or equipment, blurred work boundaries, and ineffective or inefficient communication. Especially, the ineffective communication is one of the challenges when employees work from home (Wang et al., 2021). All of these factors can be seen as a decrease of structural or social resources that employees can influence by choosing their location of work. Adaption often seems to be necessary, if unpredicted disruptions of the work processes occur (e.g., questions of colleagues and disruption of the daily schedule). Adaption also takes place in the form of adjusting to working from home and getting used to the situation. The process of selecting one of the workplaces, working hours, and work tasks is closely linked to the job crafting strategies explained by the JD-R model. By choosing one of the workplaces, employees simultaneously decide, e.g., to increase their structural or social resources. They may go to the office to discuss issues with colleagues or use the office printer, while they can choose to work from home if they want to focus on demanding tasks such as travel expense accounting.

Stempel and Siestrup (2022) suggest that the working-from-home situation might have resulted in a decline in job crafting activities because participants in their study reported fewer interruptions and less overtime, which can be seen as reduced demands. Simultaneously, the increase in autonomy was used to seek mostly structural resources and social job resources to reduce emotional exhaustion and enhance work engagement. A lack of communication and an inadequate work environment hindered crafting activities, suggesting that autonomy as a resource may not be enough to buffer these job demands. In relation to the findings by Stempel and Siestrup (2022), we found the same job crafting strategies that were applied by the interviewed employees. Insufficient resources regarding technical equipment and/or communication during working-from-home periods may have hindered crafting strategies that relied on other people. In contrast, the findings also show indications that employees have strategies to contact colleagues at the office, e.g., if work material needs to be provided. Furthermore, the combined framework of JD-R theory and time-spatial demands fit allows us to draw some initial conclusions for hybrid work arrangements. It may be that the job crafting strategies were not completely hindered by working from home but adapted according to the work space.

Previously, job crafting strategies that reduced demands were often regarded as not useful since they also decreased positive outcomes and could result in less challenging jobs and lower work engagement (Petrou et al., 2015). In the context of the COVID-19 pandemic with high autonomy provided for employees who work from home, this may not necessarily be the case. Employees may experience autonomy as a challenging job resource with positive outcomes (de Bloom et al., 2020), while the decrease in hindering job demands can function as a protective health factor and is associated with lower workaholism (Nissinen et al., 2022). Therefore, when job demands are hindering, the optimization of job demands can yield positive outcomes (Demerouti and Peeters, 2018) and could be seen as a successful strategy when working from home during the COVID-19 pandemic.

**2. How do public service employees perceive job satisfaction and productivity when working from home?**

Employees' choices regarding workplaces may impact their work satisfaction and productivity. Accordingly, interviewees in our study reflected on the advantages and disadvantages, when they chose to work from home. Other studies have found that telework can have a positive impact on work satisfaction (Hornung and Glaser, 2009; Troup and Rose, 2012; Bae and Kim, 2016) and performance (Vega et al., 2015) but that it can also increase stress in turn leading to a loss of productivity (Wu and Chen, 2020). From employees' perspectives, social interactions and advantages of working from home, particularly the acquired work autonomy, were mentioned as factors for satisfaction. Thus, employees actively select crafting strategies to enhance social exchange—either on-site or when working from home. The selection to work on-site seemed useful if social exchange or support from colleagues was needed. Concurrently, working on-site imposed more distractions and interruptions on employees and may have negatively impacted their productivity. Therefore, employees actively selected working-from-home days to concentrate on their tasks. Productivity may have also increased because employees could optimize their use of structural resources that were available at the office and at home. These results are in line with the outcomes of other studies, where job crafting activities were positively linked to performance (Boehnlein and Baum, 2020; Petrou and Xanthopoulou, 2020) and work satisfaction (Boehnlein and Baum, 2020). The results by Liu et al. (2021) suggest that job crafting could enhance performance in the context of telework. The qualitative findings of our study suggest that job crafting may be useful in hybrid work settings and allow employees to craft their productivity level by choosing a suitable workplace according to their work tasks.

All in all, employees reported dissatisfaction mainly in relation to the social distancing measures due to the COVID-19 pandemic and perceived greater productivity when working from home. Similarly, decreasing hindering job demands and performance have been positively linked, when employees increased social and structural job resources at the same time (Petrou and Xanthopoulou, 2020). The enhanced productivity may also have derived from working according to one's own rhythm because breaks could be taken flexibly. In the same regard, job crafting strategies have been found to decrease emotional exhaustion (Stempel and Siestrup, 2022).

### 5.1. Strengths and limitations

Some limitations need to be considered when interpreting the current research findings. First, there are limitations based on the nature of the qualitative study approach. The sample size and research intentions of qualitative studies do not strive to achieve external representativity. Additionally, the interviews took place at a specific time point and therefore only captured the views of public service employees working from home 1 year and a half after the beginning of the COVID-19 pandemic. The interpretation and quality of qualitative data are grounded on the subjective understanding of the researchers, which may differ from the intended interviewees' perspectives. Second, there are limitations referring to the selected sample of public service employees. Most of the interviewed participants were over the age of 50 years. The age and public service background have to be considered since the job crafting strategies may vary in other agencies or fields and for other age groups. The interviewees volunteered for the study and already participated in a prior quantitative survey. Hence, there might have been a selection bias regarding the interview partners, namely, selecting persons who preferred working from home and were especially interested in the study topic. Moreover, the limitation of the study lies in the fact that only 12 participants were interviewed, which results in a conceptual constraint as not all potential job crafting strategies and mechanisms could be thoroughly explored.

As Gibbert et al. (2008) point out, internal validity and construct validity are a precondition for external validity. While a case study does not allow for statistical generalization (Yin, 2003), a cross-case analysis with four to 10 case studies can provide a basis for analytical generalization (Eisenhardt, 1989). Therefore, the strengths of the case study are based on the measures taken to achieve internal validity and construct validity (e.g., theory triangulation and review of transcripts and codes). One strength of the study is the close orientation to the existing literature on job crafting strategies (including the time-spatial demands fit) and the JD-R model (including optimizing demands), which adds to the body of theory building. Our findings contribute knowledge of job crafting strategies in the context of working from home during the COVID-19 pandemic. Theory triangulation was used and identified patterns that could be matched to prior research. The compliance with qualitative reporting criteria and the participation of three researchers in the study may limit the abovementioned subjective bias and increase data credibility. According to the consolidated criteria for reporting qualitative research, the characteristics of the interviewers and possible biases and assumptions have been reported. Furthermore, the semi-structured interview guideline and literature-based data analysis supported a standard procedure serving construct validity. To the best of our knowledge, this was the first qualitative study to explore the insights of public service employees' job crafting strategies in the context of COVID-19 in Germany. The findings provide an integrative view of how employees combine job crafting strategies to cope with demands and increase resources. Furthermore, it adds to the growing research by exploring two relatively new job crafting strategies such as time-spatial demands fit and optimizing demands.

### 5.2. Implications for further research

Our findings suggest specific job crafting strategies that were applied by public service employees while working from home. Nevertheless, the results do not provide insights into the crafting motives of employees. Further research could evaluate if the provided autonomy due to working from home or the impact of job demands was responsible for employees engaging in job crafting activities. According to the categories of the time-spatial demands fit by Wessels et al. (2019), further research separating the process from reflection to selection and the belonging sub-categories (reflection, selection, and adaption) seems appropriate. Furthermore, we linked the resource-based crafting strategies to the time-spatial demands fit. Hence, this study adds to the notion that employees combine different job crafting strategies (Mäkikangas, 2018) and that optimizing demands might be a useful job crafting strategy (Demerouti and Peeters, 2018). However, these findings relate to the context of working from home 1 year after the outbreak of the COVID-19 pandemic. Thus, additional testing of the theoretical framework and the transferability of results to other groups of employees or even sectors should be investigated using quantitative methods. Furthermore, prior studies have found that job crafting has the potential to impact employees' productivity and satisfaction (Tims et al., 2013; Boehnlein and Baum, 2020; Petrou and Xanthopoulou, 2020; Liu et al., 2021). Future research could determine to which extent employees choose one work form over the other and how the choice affects job performance, work–life balance, or job satisfaction.

Additionally, researchers may consider employees' adaptability to see in which way it influences job crafting activities (Wang et al., 2017). It could be of interest how collective crafting may influence the choice of workplace and the applied job crafting strategies. As Hu et al. (2019) found, people can use each other's resources to strengthen their own operations. Especially, when hybrid work settings in future prevail, this might be helpful to strengthen cooperation in work teams that are not at one workplace. However, informal job crafting appeared to be more effective in strengthening work engagement than job crafting that was provided by the employer (Hu et al., 2019). Thus, the role of employees' individual crafting strategies remains important. According to Wrzesniewski et al. (2003), supervisors who provide autonomy for employees can establish a culture of job crafting. Other settings with a high job crafting culture could be explored in larger case studies to achieve theoretical saturation concerning the categories of the time-spatial demands fit and relationships between job crafting strategies. In addition, larger samples and longitudinal designs could aim to gain a profound understanding of these relationships. Other research questions that might be of interest are related to the context (e.g., organizational structure, supervisor support, and organizational culture), in which job crafting strategies can take place when employees work from home.

### 5.3. Implications for practice

For practitioners, several implications can be derived from this study. First, public service agencies could support the reflection, selection, and adaption process of employees, e.g., by providing a handbook with reflective questions to wisely select the right workplace for the task. Second, as interventions at the workplace can improve outcomes such as wellbeing, job crafting interventions can be implemented that have been successful in promoting work engagement and performance (van den Heuvel et al., 2015; van Wingerden et al., 2017a,b). Additionally, employers could offer workshops that increase employees' skills such as time management or self-discipline to work effectively with the given demands and resources. Similarly, Wang et al. (2021) identified self-discipline as a personal resource in remote work settings. Third, job autonomy is a necessary precondition for employees to be able to craft their jobs. Therefore, employers should provide employees with enough autonomy to make changes to their jobs (Harju et al., 2021). Accordingly, leaders should be informed and trained to be able to facilitate a work environment and work culture, where employees are encouraged to craft their jobs (Kniffin et al., 2021). Since seven of the interviewed participants were leaders in this study, the interviewed leaders already demonstrated a conscious handling of job autonomy. The results suggest how employees craft their jobs and which job demands and resources they approach during their crafting. They can function as possible starting points to inspire HR practices to support employees' job crafting needs and activities. For future workplace designs, the job crafting potential should be considered in discretionary HR practices (Luu, 2021). Since employees contemplated the advantages and disadvantages of working from home and the office based on the available resources, the selection process and job crafting strategies were chosen accordingly. Therefore, it is important to leave employees with the freedom of choice and flexibility to make use of their resources and adjust their crafting strategies in practice.

## 6. Conclusion

This qualitative case study enriches research on job crafting by adding specific job crafting strategies applied by public service employees 1 year after the outbreak of the COVID-19 pandemic. This was achieved using a combined theoretical framework of resource-based job crafting derived from the Job Demands–Resources model and time-spatial job crafting. Findings reveal that employees developed personal crafting strategies to cope with hindering job demands, such as work-related availability or interruptions, and to optimize working conditions by using job resources. Strategies included initiating meetings with colleagues, installing additional work equipment, and planning office and working-from-home days. The results suggest a close link between the optimization of job demands and time-spatial demands fit, leading to the best subjective fit and optimal work environment. Finally, the study provides indications for research and practice on how employees can enhance their work satisfaction and productivity through crafting activities when working from home in the post-pandemic world.

## Data availability statement

The datasets presented in this article are not readily available because the complete interview transcripts are not publicly available due to ethical and legal restrictions, as participants of this study did not agree for their full interview transcripts to be shared publicly. Requests to access the datasets should be directed to laura.seinsche@uk-koeln.de.

## Ethics statement

The studies involving humans were approved by Ethics Committee of the University of Cologne. The studies were conducted in accordance with the local legislation and institutional requirements. The participants provided their written informed consent to participate in this study. Written informed consent was obtained from the individual(s) for the publication of any potentially identifiable images or data included in this article.

## Author contributions

LS and KS contributed to the conception and design of the study and performed the content analysis. LS, KS, and JN administered the project and carried out the investigation. LS wrote the first draft of the manuscript and prepared the visualization. HP supervised the project. All authors contributed to the manuscript revision, read, and approved the submitted version.
