# Supplementary material for: Do I want to work from home today? Specific job crafting strategies of public service employees working from home during the COVID-19 pandemic in Germany: a qualitative study
Source: Front Psychol. 2023 Oct 13;14:1183812. doi: 10.3389/fpsyg.2023.1183812 (PMC10613060; doi:10.3389/fpsyg.2023.1183812)
Supplement: Supplementary file 3 [file Data_Sheet_3.PDF]

Main category	1st order sub-categories	Exemplar quotes	2nd order sub-categories	Exemplar quotes
**Reflection**	• **more scheduled meetings when WFH**	*"And it's just so easy to carry out these digital appointments. I think that if you were present, you would have fewer appointments because you might have to travel or go to another building or something. But you can arrange these appointments quite easily via digital communication channels."* (interview 9)		
	• **different day structure when WFH**	*"I have a bit of a stomachache when I think about the possibility of having to work four days a week on site again, because that would mean a complete restructuring of everyday life for us or for me as an employee. And that goes hand in hand with a different work-life balance."* (interview 9)		
	• **productivity/ concentration when WFH**	*"If I were sitting in the next room, she would also come in and simply ask. Of course, that also means that you have fewer conversations, fewer contacts. This allows you to work with more concentration, which is an advantage."* (interview 3)		
	• **preparation/ daily schedule**	*"Whereas I have also missed the time a little bit before I had the dog, because it was always something on the way to work, that you could concentrate a bit on the work already, so that you have mentally processed or prepared for it a little bit. But that's what I have now when I go out with the dog."* (interview 2)		
	• **barriers to leadership tasks when WFH**	*"If I had been on the spot, I could have intervened and said "Stop, that's not your job". (...) Since I was not there, I did not know what they were doing."* (interview 2)<br><br>*"But you can try to intervene at distance, when you notice that there is tension between two (employees) and you do not want to alienate either of them. "* (interview 2)		
	• **different relationships at the office**	→	▪ **social interaction**	*"Of course, it's not the same as when I come into the office in the morning and greet my colleagues in person and you may run into each other and use this opportunity to talk about something on the fly."* (interview 10)
		→	▪ **social exchange**	*"I mean of course face-to face you speak more small talk or something and one simply also has the body language that you see. But that's only natural."* (interview 9)<br>*"I have the feeling that I don't hear as much from people as I did when I was there (at the office)."* (interview 3)
		→	▪ **disruptions by colleagues**	*"It would be better to say, "Please make an appointment," but (laughs), yes, and that means I'm much more disturbed in my work processes."* (interview 3)
	• **advantages WFH**	→	▪ **saving commute time**	*"So there was a driving distance of one hour alone. I always went by public transport and it took me almost two hours a day to get there."* (interview 11)
		→	▪ **environmental aspects**	*"Yes, that's working from home, which is also infinitely important from the point of view of climate or climate policy. This is really another revolution. Because if we now see that a third of people are constantly working from home, that also means a third less office space. Building management is the most damaging thing to the climate."* (interview 7)
		→	▪ **closeness to familiar environment**	*"I experienced a great personal advantage, since during this Corona period I was able to watch my grandson grow up close to me. (...) We saw each other almost every day."* (interview 1)<br>*"...so that you save the way there, that it can't actually happen to you that you have forgotten something at home and you have to go back again."* (interview 5)
		→	▪ **omission of commuting**	*"And what I really like about working from home actually now, especially in winter, in the gray season, is the flexibility, that commuting is eliminated or at least partially eliminated. Thus, I can react more flexibly at the point. If it rains, I don't have to get on my bike and go to the office, but I can work from home."* (interview 8)
		→	▪ **money saving**	*"That one is in his usual environment, that also saves time and money, specifically."* (interview 1)
		→	▪ **other**	*"...or somehow has to dress especially nicely or something. Well, that's not so much the case with us now anyway, but it still makes a difference."* (interview 5)
**Selection**	• **selection of work space**	*"There are now alternating workstations. I have to use an app to book a workstation for the days I want to be in the office."* (interview 4)<br><br>*"And for me personally, I used the working from home option only very sporadically at the beginning, on one or two days a week, because it was simply a matter of using a private computer at home, sitting at the kitchen table, and all the problems that I'm sure you're familiar with and that a lot of people encountered, until the technical equipment was ready. That was the crucial point, does the equipment fit."* (interview 8)		
	• **selection of working hours**	*"And I think that's also a great advantage of using such flexibility. In the past, if we wanted to use such things, I had to go home early or come in later or something similar, and I can do that when I work from home and then still continue working."* (interview 10)<br><br>*"I logged off for an hour, then at 4 o'clock I simply resumed duty for an hour or an hour and a half."* (interview 1)		
	• **selection of work tasks**	→	▪ **tasks done in the office**	*"If there were just a few people or no one was there, I went to the office and then I just forwarded the mails home."* (interview 3)<br><br>*"There are a few tasks that you can only do in the office. For example, signing off on invoices and the like, which we don't do electronically yet."* (interview 5)
		→	▪ **tasks done WFH**	*"I would also want to strive for that (working days from home) in the future, simply because I can sometimes do certain activities better at home, because I might have fewer disruptions there than in the office, especially when the office is busy again, and I can then actually follow my own rhythm more easily."* (interview 10)
**Adaption**	→	*"I'm much more disturbed when I work at the office, right? I also make my daily schedule, but then I can't keep to it in part because I'm disturbed."* (interview 3)<br><br>*"And that's already difficult, it's all blurred up here now. From morning to night. The business and the private. And that's something you have to get used to."* (interview 1)		
**Job demands**	• **increasing challenging job demands**	→	**no wish for increasing challenging job demands**	*"But there is not actually someone who says "you have to do this", but I'm also not the type who then says "I refuse to do this", because that has to be done as well."* (interview 5)<br><br>*"Of course the work has to be done and in my opinion I have already/ I am already working to capacity. So I don't have to look for work or something like that."* (interview 2)
	• **decreasing hindering job demands**	→	▪ **decreasing time pressuring work load**	*"At the moment, we can't do as many things in addition to our work, such as building up expertise or preparing future topics conscientiously and extensively, which we could have done if there were still four of us. So there are a relatively large number of construction sites that need to be tackled, but which are not part of our day-to-day operations. At the moment, that's just a bit of a backlog. I mean, you can't do more than work."* (interview 6)
		→	▪ **decreasing distractions**	*"I get more done at home (laughs). Because then you're no longer so tied up in the building and distracted from your work."* (interview 4)
		→	▪ **decreasing interruptions by**	*"It would be better to say, "Please make an appointment," but (laughs), yes, and that means I'm much more disturbed in my work processes."* (interview 3)
		→	▪ **decreasing work-related availability**	*"Yes, they just make appointments. Then they ask me if they have any problems and then I say "Yes, when do you have time?" and then we arrange a video conference. That's just better, because you can better schedule these meetings or with colleagues in a similar way. Or for a telephone call."* (interview 3)<br><br>*"So, yes, there are different strategies, well um, if you're constantly online and constantly responding to your e-mails, you don't really get to work. You just have to find ways for yourself, maybe to structure it a bit."* (interview 1)
		→	▪ **decreasing technical job demands by work hour delimitation**	*"That also happened to me once or twice - I don't want to say happened. That was a conscious decision, because it was simply easier for me to watch an import on Saturday, because if it had broken off, I would have had a lot more work on Monday than if I had intervened at the moment when it happened. In principle, I made it easier for myself at the beginning by switching on briefly. But that rarely happens."* (interview 6)
		→	▪ **no need for decreasing hindering job demands**	*"But when he's on the phone or something, it's so loud that I can hear it through the door. So it's simply unavoidable. But I don't feel like setting up a new study upstairs (...) and isolating myself there. I don't feel like it either. I'll put up with that."* (interview 5)

**Job resources**	**• increasing social job resources**	**▪ get help from colleagues**	*"So if now a colleague simply comes over next door, I sit here and she says, "Listen, I have a question. Can you help me?" No, that's low-threshold."* (interview 3)<br><br>*"On the one hand, to find solutions, which then actually led to the practical implementation that colleagues were called who were on site and then one said 'Access the drive, I need the following documents. Send them to me by email'."* (interview 8)
		**▪ seeking exchange with supervisor**	*"That's what I've learned over the course of time: Formulating my interests and then putting them forward, and then it works out well, yes. She responds and we find solutions and support in case of doubt."* (interview 1)
		**▪ increasing communication channels**	*"When I'm on site, there's only one empty chair opposite me, so to speak. If I am concerned about the work results or the concerns of team members, I now have any number of desired channels open."* (interview 12)
		**▪ initiating social exchange at office**	*"And people just dare more in presence, to just knock or look - I also often have the door open - and come in, than when I am sitting at home."* (interview 3)<br><br>*"There you just go to the coffee room, or you go to your colleagues, who you now unfortunately only get into the house by phone or e-mail or chat or video conference, you used to very often say 'I'll just go to my colleagues around the corner or one floor higher'."* (interview 6)<br><br>*"We quickly came back to the office voluntarily for certain things, simply to see the colleagues."* (interview 8)
		**▪ initiating digital social exchange**	*"Yes, and we've set it up now so that we meet every Tuesday at 10 am in Skype for the digital coffee break, and whoever wants to participate in it, they participate in it and whoever doesn't, they don't participate in it. So that's voluntary. And there is actually almost only private talk, which is also important to me, so not official matters."* (interview 11)<br><br>*"And that works in principle, but you always have to pick up the phone. And that's a different situation, it's much harder than walking the three steps."* (interview 8)
	**• increasing structural job resources**	**▪ planning office and WFH days**	*"Then I partly sent the documents to me by email or sometimes put them in my pocket to be able to cover the next day when working from home."* (interview 8)<br><br>*"And when I got my own office chair here (at home), it got a lot better."* (interview 10)<br><br>*"So when I see that there are larger documents that I just don't want to work on, then I go to the office and then I stay there and then I print them out, among other things, and then I also stay in the office for the day."* (interview 5)<br><br>*"I set that up when I first started telecommuting, which was early 2019. It's a room of its own. I built a huge desk myself that runs the length of the room."* (interview 11)
		**▪ self-initiated skill development**	*"But by now I can do them [software applications] up and down and it's the same as always. You have to learn to do it frequently and certainly it's a relief when we all have Teams [software] now, that seems to be quite a good program."* (interview 7)<br><br>*"I used to say, 'I'm not the kind of person who can do that, I need an orderly framework, I need the way to work so that I can say, 'Now I'm here, now I'm working, and when I close up here, I'll be gone again.' In this respect, it was a learning process for me to actually be able to structure myself."* (interview 8)<br><br>*"So I will certainly rely on it more often. That has already helped me, the literature."* (interview 11)

**Satisfaction**	**• through appreciation**	*"The office also takes care of the employees and tries to actively focus on employee satisfaction and employee health. That's a very, very high level of responsibility on the part of the employer that you can feel there. I find that very pleasant, even if I don't have to make use of it myself because I'm actually doing well, but I appreciate the fact that there are these opportunities and I like that very much, yes."* (interview 6).
	**• through advantages WFH**	*"They're happy, they're so fully motivated, and those are also the cases, for example, where the work results, productivity and also the motivation have naturally shot through the roof."* (interview 7)
	**• through joy regarding work tasks**	*"We have task expansion and that's why, so that's an exciting work environment."* (interview 1)<br><br>*"You take up this profession if you are rather extroverted and if you like to be around people and also like to speak in front of groups and the like and if you like to travel and all that. It was just the reason (laughs very lightly) for my choice of profession."* (interview 5)
	**• through work autonomy**	*"So the flexibility is fantastic for me."* (interview 11)<br><br>*"This relatively high degree of autonomy, I like that. And, um, I think that's also the most important point that I would say contributes to my satisfaction."* (interview 1)
**Dissatisfaction**	**• lack of appreciation**	*"And then we are told in our routine round that 'You all did a great job and thank you very much for it', but that was it. So in this respect (...) I don't want to say now that I miss it, so that would go too far. But I have the impression that it could be more."* (interview 8)
	**• change/ omission of work tasks**	*"And on top of that, I also have to take care of certain administrative tasks in our team in connection with certain IT issues, and I'm not particularly happy about that. But that only has a limited connection with working from home. It's simply a shift of work tasks due to Corona, yes, where you then have no reason to postpone such things, because you don't have to go on an important business trip as an alternative, do you?"* (interview 5)
	**• lack of social exchange**	*"Yes, one thing that I like about my work, but which is actually very limited now due to the pandemic, is the personal contact with colleagues in the various federal states. Or even internationally, because all of these face-to-face meetings have actually all been virtual conferences in the last two years. That is something that really makes up a large part of the attractiveness of the work, which has been lost as a result. That has to be said quite clearly, yes."* (interview 10)
**Productivity when WFH**	**• focus on tasks**	*"But I can't concentrate on technical aspects of my work there, I can do that much better at home, so that's also a huge advantage."* (interview 11)
	**• due to better energy management**	*"Because I simply do some exercise at lunchtime and I somehow have the feeling that after lunch or after a lunch break, my brain switches off automatically, which is a bit of an exaggeration, but when I work from home I really start somehow fresher into the second half of the work day after lunch break."* (interview 9)
	**• due to increased working hours**	*"And then it was often the case, that this saved working time was instead converted into office work, i.e. real work in front of the computer. In other words, I worked more. Whether I was always necessarily more effective then is another question, but purely from the time and I think also from the tasks it has been more."* (interview 8)
	**• due to elimination of interruption /distraction**	*"Two days a week in any case (wish to work from home). I would also want to strive for that in the future, simply because I can sometimes do certain activities better at home, because I might have fewer disruptions there than in the office, especially when the office is busy again, and I can actually follow my own rhythm then."* (interview 10)<br><br>*"Because personally I'm quickly distracted by phone calls from my colleague or even if someone from the office comes in or something, so you're interrupted in the flow of work. That bothers me or that's definitely one of the differences between working from home and working on site. This really concentrated work for a longer period of time, reading a longer text or something (...) And that's really good for me here when I work from home, that I can simply concentrate on my work for longer periods of time, which is very difficult to do on site, yes."* (interview 9)
	**• ommission of certain tasks**	*"That's all been ommitted for 20 months now, and of course that means no more downtime, no more travel time, and no more preparation time for meetings, right? You always had to make coffee for your colleagues and prepare the room. So that's more efficient."* (interview 6)
	**• increased communication on interim results**	*"Then we might have another meeting in the middle of the day or at the end of the day, where we say "This is the work progress now. Does this fit into the management concept?" and if it doesn't fit or if it fits, then it's good, then (...) efficiency increases and if it doesn't fit, efficiency also increases, because the misguided development hasn't continued over 14 days."* (interview 12)
